# Clinical Lectures on Diseases of the Eye

**Published:** 1866-05

**Authors:** E. L. Holmes

**Affiliations:** Lecturer on Diseases of the Eye in Rush Medical College, and Surgeon to the Chicago Charitable Eye and Ear Infirmary


*Clinical Lectures on Diseases of the Eye.* By E. L. Holmes, M. D., Lecturer on Diseases of the Eye in Rush Medical College, and Surgeon to the Chicago Charitable Eye and Ear Infirmary.

### MYDRIASIS, MYOSIS, SPASM AND PARALYSIS OF THE ACCOMMODATIVE MUSCLE AND ASTIGMATISM.

Gentlemen,—Mydriasis is characterized by a marked dilation of the pupil, without any apparent abnormal changes in the tissues of the eye. In typical cases, the pupil of one or of each eye becomes dilated to such an extent, that the iris is scarcely perceived behind the limbus conjunctivæ. Sometimes the dilated pupil is oval or irregular; it is almost entirely if not quite uninfluenced by the strongest light, or by fixing the eyes upon near objects. Mydriasis is often, perhaps usually, accompanied by paralysis of the ciliary muscle, so that the eye cannot accommodate its focus for vision of near objects. In some cases the ciliary muscle is unaffected, as can be shown by placing an aperture in a thin disc of metal before the pupil, to exclude the undue amount of light which enters the dilated pupil, when it will be found that the eye can see near objects as well as distant objects. The affection is usually confined to one eye. To such an eye, objects often seem smaller than they really are. This apparent diminution in the size of objects, the dazzling caused by too much light entering the eye, and usually the inequality in the foci of the two eyes, produce the chief inconvenience of this disease. The causes of mydriasis are often so obscure, as to evade the most careful scrutiny.

Paralysis of the branches of the third pair of nerves which supply the annular fibres of the iris, or irritation of the sympathetic nerves, which supply the radiating muscles, may produce the disease. In these cases the primary cause may be masturbation, irritation of the bowels from the presence of worms or crude substances. Belladonna and other drugs taken internally or applied near the eye produce, as you well know, dilation of the pupil. The diagnosis between mydriasis, produced by belladonna and that caused by other agents, is not only difficult but sometimes impossible. Malingering soldiers, who have been taught the effects of atropine, may long keep up deception if they are cunning enough to conceal the drug from their surgeons. Soldiers have been known, for instance, to conceal some form of belladonna under the toe-nail, or in a small tube attached to a thread and introduced into the rectum.

The prognosis in cases of mydriasis is generally favorable.

In regard to treatment there is but little to be said with certainty.

It is of great importance to seek the cause and endeavor to remove it.

Extract of calabar bean has been in some cases known to act favorably in causing the pupil to contract. In other cases it has proved inert.

From the fact that the pupil contracts from irritation of the fifth pair of nerves, as in certain ulcerations of the cornea, or when foreign substances are introduced under the lids, some practitioners have been led to produce minute ulcerations artificially, by the application of solid nitrate of silver to the conjunctiva near the cornea. Occasionally, this plan has seemed successful.

Electricity, strychnia, ergot, methodical exercise of the internal recti muscles, in fixing the eyes upon near objects, have been found beneficial.

*Myosis* is that condition of the eye, in which the pupil remains for a greater or less period abnormally contracted, without apparent change in the internal structures of the eye. The pupil may be exceedingly minute, and almost wholly uninfluenced by atropine, or by absence of light. As little light can enter the pupil, the impression on the retina is diminished, and objects appear less brilliant than when the pupil is of its normal size.

Irritation of the third pair of nerves, paralysis of the sympathetic branches of the iris, habitual use of the eyes upon near objects, as with jewelers and engravers, constipation, tumors in the neck pressing upon the sympathetic nerve, have been recognized as causes of myosis. The exact condition of the accommodative apparatus has not been accurately determined.

You are well aware that the calabar bean has been found, when applied to the healthy eye, to produce extreme contraction of the pupil, and also to render the eye myopic.

The treatment of this disease consists almost entirely in removing its cause. Atropine is almost useless.

If the contraction is excessive and other treatment hopeless, the operation for artificial pupil is indicated.

Paralysis of the ciliary muscle is characterized by the impossibility of accommodating the eyes for near objects. The distance, however, at which objects can be seen, will be different in different eyes, according as they are short-sighted or hypermetropic. The paralysis may be partial or total, and usually depends upon diseases of the brain or spinal cord, upon uræmia, diabetes, lead poison and excessive use of alcohol. It is usually attended with dilatation of the pupil.

The influence of belladonna in producing paralysis of the accommodative muscle, has already been described in a previous lecture. The treatment of this disease must be conducted upon the same general principles which guide us in the management of other forms of paralysis. Much had been expected from calabar bean, although little has been really accomplished by the remedy.

Spasm of the ciliary muscle produces an effect just contrary to that of paralysis. The accommodative apparatus is spasmodically adjusted for the nearest object it is capable of seeing distinctly. This is often a result of irritation of the cornea and conjunctiva, as in certain forms of inflammation, and is accompanied with spasm of the orbicularis and of the annular fibres of the iris, and with photophobia. Occasionally it is observed without these symptoms.

The treatment of this affliction is often unsatisfactory. If possible, irritation should be removed. Exercise of the eye with the use of convex lenses, is sometimes of benefit. Since a convex lens placed before the eye, when adjusted for near objects, tends to bring the focus of the rays of light in front of the retina, there will be an effort on the part of the adjusting apparatus to overcome the effects of the conevx lens, by flattening the crystalline lens. This can only be accomplished by a relaxation of the ciliary muscle. Belladonna tends to overcome this spasm, but is often employed without any good results.

Mydriasis, myosis, paralysis and spasm of the ciliary muscle, are comparatively rare diseases. I have merely alluded to the principal facts connected with them, because they may occasionally come to your notice, and especially because these pathological conditions serve to enlighten us very materially in reference to the physiology of accommodation.

The older ophthalmologists were in great doubt regarding the power of accommodation. Many believed it depended upon the action of the recti muscles.

We have seen that paralysis of the accommodative apparatus, whether idiopathic or caused by belladonna, spasm of the accommodative apparatus, whether idiopathic or caused by calabar bean, produce respectively such changes that the eye is adjusted to distant or near objects only—the muscular fibres are unable either to contract or relax respectively. There is an analogy between these two conditions, and paralysis and spasm of the flexor muscles of the limbs. These different pathological conditions of the adjusting apparatus may exist, and yet the recti muscles may rotate the eyes as freely as in perfect health, showing that accommodation is independent of the recti muscles. You remember that I called your attention in a previous letter to the fact, that total paralysis of the recti muscles could exist without destroying the power of accommodation.

*Astigmatism.* In the normal eye the surfaces of the cornea and crystalline lens possess almost absolutely the same convexity in their horizontal, perpendicular and intermediate diameters, (Meridians.)

In certain cases the cornea, and less frequently the lens, is much more convex in one diameter than it is in another. This condition is termed *astigmatism.* The surface of the cornea is ellipsoidal. It was quite well described years ago. Donders and others, however, have investigated the subject more fully. We now find it embraces quite a long and interesting chapter in ophthalmology. When astigmatism exists in a marked degree, there is great indistinctness of vision. A square or a circle will appear respectively oblong or elliptical. As you can readily understand, if the eye is adjusted in such a way that the most convex portion of the cornea causes the rays to come to a focus *on* the retina, the less convex portion must come to a focus *behind* the retina. Hence, there will be this form of distortion in the appearance of objects, as, for instance, a square: The two opposite sides will appear parallel and quite distinct, while the other two sides will be ill-defined and irregular.

To such a degree are some eyes astigmatic, that a luminous point appears as a line.

The diagnosis of this condition of the eye usually rests upon the following modes of examination: The patient should be directed to look at some suitable object, such as a white square or circle on a dark ground, placed at a convenient distance. The character of the distortion will lead one to suspect the presence or absence of astigmatism.

If a thin disc of metal, in the centre of which a very narrow slit not more than one-half a line in length is made, be placed close to the cornea, it will be found, on looking through this opening and rotating it, that there will be a certain position of this opening in which vision will be most distinct, and when turned 90° further, vision will be most indistinct.

An examination with the ophthalmoscope is of service in the diagnosis of this deformity, since the rays of light, on passing through the astigmatic cornea, changes the apparent form of optic papilla from that of a circle to that of an ellipse. It should, however, be remembered, that the papilla is sometimes found elliptical in health.

Astigmatism may be congenital or acquired. It is usually most apparent in the cornea, although it may be found in the lens.

The causes of acquired astigmatism are chiefly ulcerations and injuries of the cornea, which on healing produce contraction of the tissues and consequent inequalities of curvature.

The extraction of cataract is sometimes followed by sloughing of the cornea. Cicatrization often results in more or less differences of curvature.

The treatment of astigmatism consists almost entirely in the use of concave or convex cylindrical lenses. By means of these lenses vision may be very much improved, and in favorable cases even restored to normal distinctness.

The lenses are most applicable in regular astigmatism, where the surface is ellipsoidal. In irregular astigmatism, where the cornea is, for instance, conical, or where its surface has a curvature similar to the surface of an egg, divided parallel to its long diameter, they will, at best, but partially improve vision.

